# Parenteral lidocaine for treatment of intractable renal colic: a case series

**DOI:** 10.1186/1752-1947-5-256

**Published:** 2011-06-29

**Authors:** Hassan Soleimanpour, Kamaleddin Hassanzadeh, Dawood Agha Mohammadi, Hassan Vaezi, Robab Mehdizadeh Esfanjani

**Affiliations:** 1 Emergency Medicine Department, Tabriz University of Medical Sciences, Daneshgah Street, Tabriz 51664, Iran; 2 Urology Department, Tabriz University of Medical Sciences, Daneshgah Street, Tabriz 51664, Iran; 3 Anesthesiology Department, Tabriz University of Medical Sciences, Daneshgah Street, Tabriz 51664, Iran; 4 Department of Statistics, Tehran North Branch, Islamic Azad University, Tehran, Iran

## Abstract

**Introduction:**

We report a case series of successful treatment of intractable renal colic using parenteral lidocaine.

**Case presentation:**

Because of inconsistent responses to standard treatment with opioids and non-steroidal anti-inflammatory drugs in patients with renal colic pain, we decided to begin a trial of a single intravenous dose of lidocaine (approximately 1.5 mg/kg) slowly in eight patients with intractable renal colic who were referred to our emergency medicine department. The patients were six men and two women with a mean age at diagnosis of 34.62 years (age range, 28 to 42 years). The patients were of Iranian ethnic origin. The patients' degree of pain, based on Visual Analog Scale score upon entering our emergency medicine department, was recorded 10, 20, and 30 minutes after lidocaine injection. The patients' degree of pain decreased from a mean Visual Analog Scale score (±SD) of 8.87 ± 0.99 (95% confidence interval (95% CI) 8.04 to 9.70) to a mean Visual Analog Scale score (±SD) of 1 ± 2.82 (95% CI -1.36 to 3.36) before and 30 minutes after lidocaine treatment, respectively. Two of eight patients experienced transient mild dizziness, and three of eight patients experienced minimal slurring of speech. No patient experienced serious adverse events.

**Conclusion:**

Parenteral lidocaine treatment can reduce pain dramatically or subtly.

## Introduction

Numerous medications have been utilized for pain relief in patients with renal colic; however, no medication has yet been found to relieve pain quickly and completely. A variety of drugs are used to treat this condition, including anti-spasmodics, non-steroidal anti-inflammatory drugs (NSAIDs), and opioids [1]. Parenteral lidocaine has been reported to be effective in small studies of various neuropathic pain conditions, including diabetic neuropathy, post-operative pain, post-herpetic neuralgia, centrally mediated pain, headache, and malignant nerve infiltration [2-5].

## Case presentation

Eight patients (mean age (±SD) 34.62 ± 4.34 years, six men and two women) with intractable renal colic who presented to our emergency department were enrolled into this study. All included patients previously underwent therapy with morphine and NSAIDs previously but were resistant to treatment. Adults with acute flank pain radiating to the groin, vomiting, and urinary symptoms were eligible for inclusion in the study. Every aspect of the present study was explained to the patients, and we obtained their written consent for participation in the study. The diagnosis of renal stone was confimed by ultrasonsgraphy and by the presence of red blood cells (RBCs) revealed by urinalysis [6,7]. Because of patients' inconsistent responses to standard treatment with opioids and NSAIDs for renal colic pain, the decision was made to begin a trial of a single, slowly administered intravenous dose of lidocaine (approximately 1.5 mg/kg) for these patients. As fluid therapy is not commonly used in our center to treat patients with renal colic and is used only for patients who are dehydrated or have sepsis, our patients received very little fluid (10 mL) administered only by injection [8]. The mean pain degree reported by our patients was recorded based on Visual Analog Scale (VAS) score before and 10, 20, and 30 minutes after injection (Table 1). The mean VAS scores (±SD) decreased from 8.87 ± 0.99 (95% confidence interval (95% CI) 8.04 to 9.70) to 1 ± 2.82 (95% CI -1.36 to 3.36) before and 30 minutes after receiving lidocaine treatment, respectively. Colic pain disappeared completely within 10, 20, and 30 minutes in four of eight, six of eight, and seven of eight patients, respectively. The vital signs of the patients did not change during the 30 minutes. Two of eight patients experienced transient mild dizziness, and three of eight patients experienced minimal slurring of speech. No patients experienced serious adverse events. One patient needed another anodyne treatment after 30 minutes. For further studies, a kidney, ureter, and bladder (KUB) X-ray was requested immediately for the patient who was resistant to lidocaine. However, for the other patients who had responded to lidocaine treatment, KUB X-rays with previous preparation were requested, and the results are presented in Table 2. Complete pain relief was reported in seven of eight patients after lidocaine administration, and this lasted until hospital discharge. These patients were followed up by telephone for the following 24 hours. To reduce the risk of stone recurrence, all patients were instructed to force oral hydration to produce at least 2L/day urine after discharge from the emergency room. Six of the patients reported that their renal colic had not come back during the previous 24 hours, but one patient who experienced renal colic rated his pain as 3 of 10 on the VAS. His pain was relieved by NSAID therapy.

**Table 1:** Visual Analog Scale score at each 10-minute interval^a^

	Before treatment	10 minutes after treatment	20 minutes after treatment	30 minutes after treatment
Mean VAS score ± SD	8.87 ± 0.99 (95% CI 8.04 to 9.70)	1.75 ± 2.18 (95% CI -0.07 to 3.57)	1.25 ± 2.81 (95% CI -1.10 to 3.60)	1 ± 2.82 (95% CI -1.36 to 3.36)

^a^VAS, Visual Analog Scale; SD, standard deviation; 95% CI, 95% confidence interval.

**Table 2:** Individual patient characteristics

Case	Age, years	Gender	Kind of Stone	Stone side	Stone condition	Hydronephrosis
1	31	F	Radiopaque	Left	RS	Moderate
2	32	M	Radiopaque	Right	FS	Moderate
3	38	M	Radiopaque	Left	RS	Mild
4	28	F	Radiolucent	Right	FS	Mild
5	42	M	Radiopaque	Left	FS	Moderate
6	35	M	Radiopaque	Left	FS	Moderate
7	36	M	Radiopaque	Right	FS	Moderate
8	35	M	Radiolucent	Right	RS	Mild

RS:Recurrent Stone; FS:First Stone.

Case 1 was a 31-year-old Iranian woman who was referred to our emergency department because of refractory renal colic (resistant to morphine and NSAIDs). The patient had a history of renal colic, and, in spite of receiving morphine and NSAIDS previously, she mentioned no decrease in her degree of pain (VAS score 10 of 10). Her clinical examination revealed tenderness in her left costovertebral angle (CVA) radiating to the genitalia, which was associated with dysuria, urination frequency, and nausea. Abundant RBCs were detected by urinalysis, and moderate hydronephrosis of the left kidney was visualized by ultrasonography. After obtaining written consent from the patient, lidocaine 1.5 mg/kg was administered intravenously. Ten, twenty, and thirty minutes after lidocaine treatment, the patient's VAS scores decreased to 3 of 10, 0 of 10, and 0 of 10, respectively. After lidocaine administration, the patient experienced transient dizziness for less than one minute. The patient was followed up in the hospital until she was discharged. Follow-up was carried out by telephone for more than 24 hours after discharge. The patient experienced no colic pain during the follow-up period. On a planned KUB X-ray requested the day after her discharge from the emergency ward, a radiopaque stone was observed.

Case 2 was a 32-year-old Iranian man who was referred to our emergency department because of refractory renal colic (resistant to morphine and NSAIDs). Despite the fact that he had previously received treatment with morphine and NSAIDs, he mentioned no regression in pain degree (VAS score 9 of 10). His clinical examination revealed tenderness in his right CVA radiating to the genitalia, which was associated with dysuria, hematuria, nausea, and vomiting. Abundant RBCs were revealed by urinalysis, and moderate hydronephrosis of his right kidney was visualized by ultrasonography. After obtaining written consent from the patient, lidocaine 1.5 mg/kg was administered intravenously. Ten, twenty, and thirty minutes after lidocaine treatment, the patient's VAS scores decreased to 2 of 10, 0 of 10, and 0 of 10, respectively. The patient experienced transient dizziness and slurring of speech lasting less than one minute after lidocaine administration. He was followed up in the hospital until he was discharged. The follow-up procedure was continued by telephone for more than 24 hours after discharge. The patient did not report any colic pain during the follow-up period. On a planned KUB X-ray requested the day after his discharge from the emergency ward, a radiopaque stone was observed.

Case 3 was a 38-year-old Iranian man who was referred to our emergency department because of refractory renal colic (resistant to morphine and NSAIDS). The patient had a history of renal colic, and, in spite of receiving treatment with morphine and NSAIDs previously, he had no regression in pain degree (VAS score 10 of 10). His clinical examination revealed tenderness in the left CVA radiating to the genitalia, which was associated with dysuria, urination frequency, and nausea. Abundant RBCs were revealed by urinalysis, and mild hydronephrosis of the left kidney was visualized by ultrasonography. With the patient's written consent, lidocaine 1.5 mg/kg was administered intravenously. Ten, twenty, and thirty minutes after treatment, the patient's VAS scores decreased to 0 of 10, 0 of 10, and 0 of 10, respectively. After lidocaine administration, he experienced no adverse events. The patient was followed up in the hospital until he was discharged. His follow-up was extended until the day after discharge and was carried out by telephone. During the follow-up period, the patient reported no colic pain. On a planned KUB X-ray requested the day after his discharge from emergency ward, a radiopaque stone was observed.

Case 4 was a 28-year-old Iranian woman who was referred to our emergency department because of refractory renal colic (resistant to morphine and NSAIDs). In spite of her previous treatment with morphine and NSAIDs, she had no regression in pain degree (VAS score 9 of 10). Her clinical examination revealed tenderness in her right CVA radiating to the genitalia, which was associated with dysuria, urination frequency, and nausea. Abundant RBCs were revealed by urinalysis, and mild hydronephrosis of the right kidney was visualized by ultrasonography. The patient's written consent was obtained, and lidocaine 1.5 mg/kg was administered intravenously. Ten, twenty, and thirty minutes after treatment, the patient's VAS scores decreased to 0 of 10, 0 of 10, and 0 of 10, respectively. After lidocaine administration, she experienced transient dizziness and slurring of speech for less than one minute. The patient was followed up in the hospital until she was discharged. Follow-up was extended and carried out by telephone for more than 24 hours after her discharge. She reported no colic pain during the follow-up period. On a planned KUB X-ray requested the day after her discharge from our emergency ward, a radiolucent stone was observed.

Case 5 was a 42-year-old Iranian man who was referred to our emergency department because of refractory renal colic (resistant to morphine and NSAIDs). Although he had previously received morphine and NSAIDs, he had no regression in pain degree (VAS score 9 of 10). His clinical examination revealed tenderness in the left CVA radiating to the genitalia, which was associated with dysuria, urination frequency, nausea, and vomiting. Abundant RBCs were revealed by urinalysis, and moderate hydronephrosis of the left kidney was visualized by ultrasonography. Once the patient's written consent was obtained, lidocaine 1.5 mg/kg was administered intravenously. Ten, twenty, and thirty minutes after lidocaine treatment, the patient's VAS scores decreased to 3 of 10, 2 of 10, and 0 of 10, respectively. After lidocaine administration, the patient did not experience any adverse events. The patient was followed up in the hospital until he was discharged. Follow-up was continued by telephone for more than 24 hours after discharge. The patient reported no colic pain during the follow-up period. On a planned KUB X-ray requested the day after his discharge from our emergency ward, a radiopaque stone was observed.

Case 6 was a 35-year-old Iranian man who was referred to our emergency department because of refractory renal colic (resistant to morphine and NSAIDs). In spite of receiving previous treatment with morphine and NSAIDs, he had no regression in pain degree (VAS score 7 of 10). His clinical examination revealed tenderness in the left CVA radiating to the genitalia, which was associated with dysuria, urination frequency, nausea, and vomiting. Abundant RBCs were detected by urinalysis, and moderate hydronephrosis of the left kidney was visualized by ultrasonography. After obtaining written consent from the patient, lidocaine 1.5 mg/kg was administered intravenously. Ten, twenty, and thirty minutes after treatment, the patient's VAS scores decreased to 0 of 10, 0 of 10, and 0 of 10, respectively. After lidocaine administration, the patient experienced no adverse events. The patient was followed up in the hospital until he was discharged. Follow-up was carried out by telephone for more than 24 hours after he was discharged. The patient reported no colic pain during the follow-up period. On a planned KUB X-ray requested the day after his discharge from our emergency ward, a radiopaque stone was observed.

Case 7 was a 36-year-old Iranian man who was referred to our emergency department because of refractory renal colic (resistant to morphine and NSAIDs). He had previously been treated with morphine and NSAIDs, but he had no regression in pain degree (VAS score 9 of 10). His clinical examination revealed tenderness in his right CVA radiating to the genitalia, which was associated with hematuria, dysuria, and urination frequency. Abundant RBCs were revealed by urinalysis, and moderate hydronephrosis of the right kidney was visualized by ultrasonography. After obtaining the patient's written consent, lidocaine 1.5 mg/kg was administered intravenously. Ten, twenty, and thirty minutes after treatment, the patient's VAS scores decreased to 6 of 10, 8 of 10, and 8 of 10, respectively. After lidocaine administration, the patient did not experience any adverse events. Because of his inconsistent response to lidocaine, this patient needed additional anodyne treatment. A KUB X-ray was requested immediately for this patient for further study, and a number of radiopaque stones were observed.

Case 8 was a 35-year-old Iranian man who was referred to our emergency department because of refractory renal colic (resistant to morphine and NSAIDs). The patient had a history of renal colic, and, in spite of receiving previous treatment with morphine and NSAIDs, he had no regression in pain degree (VAS score 8 of 10). His clinical examination revealed tenderness in the right CVA radiating to the genitalia, which was associated with dysuria, urination frequency, nausea, and vomiting. Abundant RBCs were revealed by urinalysis, and moderate hydronephrosis of the right kidney was visualized by ultrasonography. Once his written consent was obtained, lidocaine 1.5 mg/kg was administered intravenously. Ten, twenty, and thirty minutes after treatment, the patient's VAS scores decreased to 0 of 10, 0 of 10, and 0 of 10, respectively. After lidocaine administration, the patient experienced transient slurring of speech for less than one minute. The patient was followed up in the hospital until he was discharged. Follow-up was carried out for more than 24 hours by telephone while he was resting at home after discharge. This patient experienced renal colic and rated his pain as 3 of 10 on the VAS scale. His pain was relieved by administration of NSAIDs. On a planned KUB X-ray requested the day after his discharge from our emergency ward, a radiolucent stone was observed.

## Conclusion

We have presented a short-lived but simple, inexpensive, low-risk, and effective technique for treating pain associated with intractable renal colic. Lidocaine is effective for treating patients with visceral or central pain [2]. Lidocaine has been hypothesized to alter sympathetic tone to the smooth muscle by suppressing transmission through afferent sensory pathways [9]. Parenteral lidocaine can reduce pain dramatically or subtly. Parenteral lidocaine is useful when opioids are ineffective or cause unacceptable adverse effects [2].

## Abbreviations

KUB: kidney, ureter, and bladder X-ray; NSAIDs: non-steroidal anti-inflammatory drugs; RBC: red blood cell; RS: recurrent stone; VAS: Visual Analog Scale; FS: first stone.

## Consent

Written informed consent was obtained from the patient for publication of this case report and any accompanying images. A copy of the written consent is available for review by the Editor-in-Chief of this journal.

## Competing interests

The authors declare that they have no competing interests.

## Authors' contributions

HS, KH, DA, and HV collected clinical data, reviewed the literature on the topic, and drafted the manuscript. RME analyzed and interpreted the patient data. All of the authors were involved in patient management or the writing of the manuscript. All authors read and approved the final manuscript.

## References

[B1] Labrecque M Dostaler LP Rousselle R Nguyen T Poirier S Efficacy of nonsteroidal anti-inflammatory drugs in the treatment of acute renal colic: a meta-analysis Arch Intern Med 1994 154 1381 1387 10.1001/archinte.154.12.1381 8002690

[B2] Ferrini R Paice JA How to initiate and monitor infusional lidocaine for severe and/or neuropathic pain J Support Oncol 2004 2 90 94 15330376

[B3] Wolff RF Bala MM Westwood M Kessels AG Kleijnen J 5% lidocaine medicated plaster in painful diabetic peripheral neuropathy (DPN): a systematic review Swiss Med Wkly 2010 140 297 306 20458651 10.4414/smw.2010.12995

[B4] Schwartzman RJ Patel M Grothusen JR Alexander GM Efficacy of 5-day continuous lidocaine infusion for the treatment of refractory complex regional pain syndrome Pain Med 2009 10 401 412 10.1111/j.1526-4637.2009.00573.x 19284488

[B5] McKay A Gottschalk A Ploppa A Durieux ME Groves DS Systemic lidocaine decreased the perioperative opioid analgesic requirements but failed to reduce discharge time after ambulatory surgery Anesth Analg 2009 109 1805 1808 10.1213/ANE.0b013e3181be371b 19923506

[B6] Marx J Hockberger R Walls R Rosen's Emergency Medicine: Concepts and Clinical Practice 2010 7 Philadelphia: Mosby 1309 131121720654

[B7] Pervez A Arif A Role of ultrasound in evaluation of renal colic and assessment of risk factor for renal calculi Gomal J Med Sci 2007 5 22 27

[B8] Purohit RS Stoller ML Wessells H, McAninch JW Renal colic resulting from renal calculus disease Urological Emergencies: A Practical Guide 2005 Totowa, NJ: Humana Press 241 262

[B9] Rimbäck G Cassuto J Tollesson PO Treatment of postoperative paralytic ileus by intravenous lidocaine infusion Anesth Analg 1990 70 414 419 10.1213/00000539-199002001-00414 2316883

